# A comparison of health-related quality of life (HRQoL) across four systemic autoimmune rheumatic diseases (SARDs)

**DOI:** 10.1371/journal.pone.0189840

**Published:** 2017-12-19

**Authors:** Julia Greenfield, Marie Hudson, Evelyne Vinet, Paul R. Fortin, Vivian Bykerk, Christian A. Pineau, Mianbo Wang, Sasha Bernatsky, Murray Baron, Canadian Scleroderma Research Group and Canadian Inflammatory Myopathy Study Group

**Affiliations:** 1 Department of Anatomy McGill University, Montréal, Quebec, Canada; 2 Medicine, McGill University, Montréal, Quebec, Canada; 3 Division of Rheumatology Jewish General Hospital, Montréal, Quebec, Canada; 4 Lady Davis Institute, Jewish General Hospital, Montréal, Quebec, Canada; 5 Division of Rheumatology, McGill University Health Center, Montreal, Quebec, Canada; 6 Division of Rheumatology, Department of Medicine, Centre Hospitalier Universitaire de Québec, Quebec City, Quebec, Canada; 7 Université Laval, Quebec City, Quebec, Canada; 8 Division of Rheumatology, Hospital for Special Surgery, Cornell University, New York, New York, United States of America; Soroka University Medical Center, ISRAEL

## Abstract

**Objectives:**

To compare physical and mental health-related quality of life (HRQoL) across four systemic autoimmune rheumatic diseases (SARD).

**Methods:**

Incident subjects enrolled in four SARD cohorts, namely systemic lupus erythematosus (SLE), systemic sclerosis (SSc), rheumatoid arthritis (RA) and idiopathic inflammatory myopathies (IIM) were studied. The outcomes of interest were baseline Short Form Health Survey physical (PCS) and mental (MCS) component summary scores. Multivariate analysis was conducted to determine whether PCS and MCS scores differed across SARD type.

**Results:**

The study included 118 SLE (93% women, mean age 36 years), 108 SSc (79% women, mean age 55), 64 RA (63% women, mean age 58) and 25 IIM (68% women, mean age 49) subjects. Mean PCS scores were 38.9 ± 12.2 in SLE, 37.1 ± 13.3 in RA, 35.0 ± 13.6 in SSc and 28.0 ± 15.4 in IIM. Mean MCS scores were 45.0 ± 13.3 in RA, 44.4 ± 14.7 in SSc, 40.1 ± 14.3 in SLE and 33.6 ± 18.7 in IIM. SARD type was an independent predictor of HRQoL with, in some cases, the magnitude of the differences reaching one standard deviation (IIM worse PCS scores compared to SLE (β -12.23 [95% CI -18.11, -6.36; p<0.001]); IIM worse MCS scores compared to SSc (β -11.05 [95% CI -17.53, -4.58; p = 0.001]) and RA (β -11.72 [95% CI -18.62, -4.81; p = 0.001]).

**Conclusions:**

Cross-SARD research provides a novel approach to gain greater understanding of commonalities and differences across rheumatic diseases. The differences observed warrant further research into correlates and trajectories over time.

## Introduction

Systemic autoimmune rheumatic diseases (SARDs) are chronic, systemic inflammatory diseases characterized by autoimmunity. Individually, SARDs are not common, but collectively they affect 5% of the population and are associated with high rates of disability, premature mortality, and significant societal costs[1, 2].Rheumatoid arthritis (RA), systemic lupus erythematosus (SLE), idiopathic inflammatory myopathies (IIM) and systemic sclerosis (SSc) are SARDs that share demographic, clinical, serological, immunological and genetic features. For example, these diseases affect women more commonly than men; common clinical features include Raynaud’s phenomenon, sicca syndrome, inflammatory arthritis and lung disease; antinuclear antibodies [ANA] are common, T and B lymphocytes play a central role [3, 4] and a common type I interferon signature characterizes SARDs [5]; and SARD have common genetic defects eg. MHC class II alleles, STAT 4, PTPN22 and IRF5 loci[6, 7]. Nevertheless, there are clear disease-specific features such as skin fibrosis in SSc, anti-CCP antibodies specific to RA and disease-specific genetic susceptibilities[8]. Research across diseases has the potential to identify mechanistic commonalities, as well as bring to light disease-specific abnormalities.

The importance of studying health-related quality of life (HRQoL) is now widely accepted (www.nihpromis.org). This reflects the recognition that for most chronic diseases there are no cures and the benefits of therapy are often limited by side effects. Thus, in addition to standard measurements of morbidity and mortality, HRQoL assessments are widely used to inform clinicians, researchers, and policy-makers on issues of patient management, research priorities and policy decisions.

Individual SARDs are relatively uncommon diseases and this has sometimes hampered research in HRQoL in specific SARD types. Thus, although there is some evidence that HRQoL is impaired in RA, SSc and SLE [9–11], the literature remains scant in early disease, in IIM and across SARDs. Given the biological and clinical similarities and differences across SARDs, research in HRQoL in SARDs could be enhanced by studying different SARDs together. Thus, there is “power in numbers” and, in addition to traditional biomedical research, cross-disease SARD research has the potential to be a valuable tool for patient-oriented research

We undertook this study to compare the magnitude of impairment in HRQoL among incident subjects in four SARD cohorts, and to determine whether SARD type had an independent effect on HRQoL.

## Materials and methods

### Design

This was a study of cross-sectional data from SARD subjects consecutively enrolled at the time of their diagnosis within four longitudinal SARD cohorts.

### Study subjects

The study subjects were members of one of four cohorts, the Canadian Scleroderma Research Group (CSRG) cohort, the Canadian Early Arthritis Cohort Study (CATCH) cohort followed at one site (Jewish General Hospital, Montreal, Canada), the McGill University Health Center Systemic Lupus Erythematosus Cohort (MUHC SLE cohort), and the Canadian Inflammatory Myopathy Study (CIMS) cohort. The details of these individual studies are available in the Supporting information (S1 File). The CATCH and CIMS cohorts are incident cohorts whereas the CSRG and MUHC SLE cohorts include incident and prevalent subjects. Thus, to ensure that the 4 samples were comparable, only incident SSc (defined as onset of first non-Raynaud’s disease manifestation < 1 year) and SLE (defined as time of appearance of a fourth classification criteria for SLE < 1 year) subjects were included in this study. This study was approved by the ethics committee of McGill University and all participating study sites. All patients signed an informed consent to participate in longitudinal data collection and sharing of data.

### Sociodemographic variables

Sociodemographic variables (age, sex, race/ethnicity, education, smoking, employment status) were self-reported by study subjects. Subjects were allowed to report one or more of the following ethnicities: White, Hispanic, Black, Asian, Aboriginal, or other. Education was divided into 3 groups (only some elementary, high school diploma, or college/trade school/university diploma) and patients were categorized according to the highest level of schooling they completed. Smoking exposure was divided into 3 categories: never, past or current.

### Disease variables

Disease duration was recorded by the study physician and defined as the time between disease onset and baseline registry visit. The presence of Raynaud’s phenomenon, inflammatory arthritis, interstitial lung disease and myositis, and exposure to corticosteroids and disease modifying anti-rheumatic drugs (DMARDs), were determined using physician reports and detailed harmonization rules to allow the variables from the 4 study cohorts to be comparable. The presence of sicca was patient-reported in the CSRG, CIMS and CATCH cohorts (but not available for the SLE subjects). Global assessments of disease activity and damage were recorded by study physicians, using either numerical rating scales or visual analogue scales ranging from 0 to 10. Of note, a physician-reported global assessment of disease damage was not available for the CATCH subjects. Details of the study variables are provided in the Supporting information (S1 File).

### Outcome variables

HRQoL was measured using the Medical Outcomes Trust Short Form 36 (SF-36) or Short Form 12 (SF-12) Physical Component Summary (PCS) and Mental Component Summary (MCS) scores. The SF-36 consists of 36 items in 8 domains: physical functioning, social functioning, role limitations related to physical problems, role limitations related to emotional problems, mental health, vitality, bodily pain, and general health perceptions. Each domain can be scored separately with scores ranging from 0 (worst health state) to 100 (best health state). Domain scores can be summarized into two summary scores, the PCS and MCS, which are normalized to a mean of 50 with a standard deviation of 10, with lower scores indicating worse and higher scores better HRQoL. The SF-12 consists of 12 items selected from the 8 domains of the SF-36 and can also be used to generate PCS and MCS scores more efficiently. The MUHC SLE cohort used version 1 of the SF-36 while the CSRG and CIMS cohorts used version 2. CATCH used version 2 of the SF-12. The PCS and MCS scores of all forms of the questionnaire have been shown to be directly comparable [12, 13].

### Statistical analysis

PCS and MCS scores were normalized to Canadian age and sex means[14].Descriptive statistics were used to summarize the baseline characteristics of the patients. Multivariate regression analysis was conducted to determine whether SARD type had an independent effect on baseline PCS and MCS, after adjusting for sociodemographic variables (age, sex, race/ethnicity and education). All statistical analyses were performed with SAS v.9.2 (SAS Institute, USA).

## Results

The study included 118 SLE, 108 SSc (49% limited, 51% diffuse), 64 RA and 25 IIM incident subjects. There were some sociodemographic differences between the four samples (Table 1), including SLE subjects who were younger (mean age SLE 36, IIM 49, SSc 55, and RA 58 years), more commonly women (proportion female SLE 93%, SSc 79%, IIM 68% and RA 63%) and less commonly white (proportion SLE 53%, IIM 68%, RA 80%, and SSc 91%). Over two thirds of the RA and SLE subjects had greater than high school education, compared to 52% of the SSc and 48% of the IIM subjects.

**Table 1:** Baseline characteristics of study subjects (*RA SF-12, SLE SF-36v1, SSc and IIM SF-36v2).

	RA (N = 64)		SLE (N = 118)		SSc (N = 108)		IIM (N = 25)	
Sociodemographic variables	Mean or %	SD or N	Mean or %	SD or N	Mean or %	SD or N	Mean or %	SD or N
Age, years	57.9	14.1	35.8	14.2	54.8	12.5	49.4	15.4
Female, %	62.5%	40	92.7%	109	78.7%	85	68.0%	17
Race/Ethnicity, %								
White	79.7%	51	53.4%	62	90.7%	98	68.0%	17
Black	1.6%	1	18.1%	21	0.9%	1	8.0%	2
Asian	6.2%	4	14.7%	17	2.8%	3	16.0%	4
Aboriginal	1.6%	1	0.9%	1	1.9%	2	0%	0
Others	10.9%	7	12.9%	15	3.7%	4	8.0%	2
Education, %								
High school or less	32.8%	21	28.4%	33	48.1%	52	52.0%	13
> High school	67.2%	43	71.6%	83	51.9%	56	48.0%	12
Full- or part-time employment, %	56.3%	36	-	-	50.9%	55	32.0%	8
Smoking, %								
Never	51.5%	33	60.7%	71	45.4%	49	68.0%	17
Current	9.4%	6	14.5%	17	12.0%	13	0%	0
Past	39.1%	25	24.8%	29	42.6%	46	32.0%	8
**Disease variables**								
Disease duration, years	0.5	0.3	0.4	0.3	0.7	0.2	0.5	0.3
Inflammatory arthritis, %	100.0%	64	70.3%	83	26.9%	29	32.0%	8
Interstitial lung disease, %	0	0	2.5%	3	30.6%	33	68.0%	17
Myositis, %	0	0	3.2%	3	13.9%	15	100.0%	25
Raynaud's phenomenon, %	3.1%	2	20.5%	24	89.8%	97	20.0%	5
Sicca, %	19.1%	12	-	-	40.2%	43	44.0%	11
Global disease activity (range 0–10)	4.2	2.3	2.5	2.6	4.7	2.8	4.8	1.9
Global disease damage (range 0–10)	-	-	0.5	1.2	4.0	2.6	1.4	1.6
**Medications variables, currently**								
Corticosteroids	23.4%	15	61.1%	44	27.3%	27	85.0%	17
DMARDS	37.5%	24	91.2%	104	26.5%	27	85.7%	12
**Outcome variables**								
Physical component summary score*	37.1	13.3	38.9	12.2	35.0	13.6	28.0	15.4
Mental component summary score*	45.0	13.3	40.1	14.3	44.4	14.7	33.6	18.7

There were some noteworthy differences in the presence of selected clinical features across diseases (Table 1). In particular, by definition, all RA subjects had inflammatory arthritis. However, extra-articular disease was generally minimal in early RA. In contrast, all IIM subjects had inflammatory muscle disease but, in addition to that, inflammatory arthritis (32%), interstitial lung disease (68%) and sicca (44%) were common. SSc subjects also had considerable systemic involvement (eg. inflammatory arthritis 27%, interstitial lung disease 31%, inflammatory myositis 14%). The most striking feature in SSc, though, was the presence of at least moderate disease damage in early disease (global disease damage 4.0±2.6 on a scale of 0–10). Inflammatory arthritis was the most common clinical feature in SLE subjects (70%).

HRQoL was significantly impaired in SARD subjects with early disease. The mean PCS scores were lowest in IIM (28.0), representing more than 2 standard deviations below the general population, intermediate in SSc (35.0) and RA (37.1) and highest, albeit still more than one standard deviation below the general population, in SLE (38.9). The mean MCS was also lowest in IIM subjects (33.6) subjects, almost 2 standard deviations below the general population. Mental HRQoL status in early SLE was moderately impaired (40.1) and only somewhat impaired in SSc (44.4) and RA (45.0).

Multiple linear regressions were performed to analyze whether PCS and MCS scores differed across SARD, after adjusting for sociodemographic variables (Tables 2 and 3). We found that SARD type was an independent predictor of physical HRQoL: IIM was significantly worse compared to SSc (β -5.78 [95% CI -11.54, -0.03; p = 0.049]), SLE (β -12.23 [95% CI -18.11, -6.36; p<0.001]), and RA (β -7.44 [95% CI -13.57, -1.30; p = 0.018]). SLE was significantly better compared to SSc (β 6.45 [95% CI 2.36, 10.54; p = 0.002]) and RA (β 4.80 [95% CI 0.05, 9.55; p = 0.048]). Impairment in physical HRQoL was comparable in RA and SSc subjects.

**Table 2:** Multiple linear regression showing the effect of disease on PCS.

	β	95% CI		p value
Age, years	0.21	0.10	0.32	< .001
Female	3.39	-0.39	7.17	0.078
White	-1.04	-4.58	2.50	0.562
Education (> high school)	1.84	-1.29	4.97	0.248
Disease				
IIM vs SSc	-5.78	-11.54	-0.03	0.049
IIM vs SLE	-12.23	-18.11	-6.36	< .001
IIM vs RA	-7.44	-13.57	-1.30	0.018
SLE vs SSc	6.45	2.36	10.54	0.002
SLE vs RA	4.80	0.05	9.55	0.048
RA vs SSc	1.65	-2.48	5.79	0.432

CI, confidence interval

**Table 3:** Multiple linear regression showing the effect of disease on MCS.

	β	95% CI		p value
Age, years	-0.04	-0.17	0.08	0.502
Female	1.26	-2.99	5.50	0.561
White	-0.89	-4.87	3.09	0.659
Education (> high school)	1.45	-2.07	4.97	0.419
Disease				
IIM vs SSc	-11.05	-17.53	-4.58	0.001
IIM vs SLE	-5.18	-11.79	1.42	0.124
IIM vs RA	-11.72	-18.62	-4.81	0.001
SLE vs SSc	-5.87	-10.47	-1.27	0.013
SLE vs RA	-6.54	-11.88	-1.19	0.017
RA vs SSc	0.67	-3.99	5.32	0.778

CI, confidence interval

Similarly, disease was an independent predictor of mental HRQoL: IIM subjects had significantly lower MCS scores compared to SSc subjects (β -11.05 [CI 95% -17.53, -4.58, p = 0.001]) and to RA subjects (β -11.72 [CI 95% -18.62, -4.81; p = 0.001]). SLE subjects had significantly lower MCS scores compared to RA subjects (β -6.54 [CI 95% -11.88, -1.19; p = 0.017]) and SSc subjects (β -5.87 [CI 95% -10.47, -1.27; p = 0.013]). Impairment in mental HRQoL was comparable in RA and SSc subjects and in IIM and SLE subjects.

## Discussion

Among incident SARD subjects, we found that disease was an independent predictor of HRQoL, with IIM subjects having the worst physical and mental HRQoL status at disease onset, SSc and RA subjects having considerably impaired physical but not mental HRQoL and SLE subjects having moderate impairment in both physical and mental HRQoL. In addition, developers of the SF-36 have recommended a 3-point threshold for a minimal important difference (MID) in PCS and MCS across groups of medical conditions[15]. Most of the differences between SARD-type in this study were well above this threshold. Thus, the differences between diseases were not only statistically but also clinically significant.

HRQoL is known to be considerably impaired in SARDs [9–11, 16]. However, there is a paucity of data in incident disease. Few studies have reported SF-36 PCS and MCS scores in early RA, with generally small samples (N = 50–78) and low study quality [11, 17–19]. Not surprisingly, results have been widely divergent (PCS ranging from 25.6–37.3 and MCS ranging from 29.5–52.7). Recently, SF-36 PCS and MCS scores in an international (North America, Europe, Asia) inception cohort of SLE subjects (N = 495) were reported to be 39.4±11.3 and 44.9±11.8, respectively, at disease onset. There is a paucity of data on HRQoL in incident SSc and in IIM. Our results are generally consistent with the published literature in early RA and SLE, and in fact contribute important novel data for SSc and IIM.

Studies to date have generally examined HRQoL in individual SARDs, compared to the general population or subjects with other chronic diseases[16]. A notable exception was a study by Johnson et al. who compared the SF-36 PCS scores of subjects with prevalent SSc (n = 34) to that of subjects with other prevalent rheumatic diseases, including SLE (n = 74) and RA (n = 42) [20]. The mean score of SSc subjects (31.8±13.7) was lower than that of SLE (39.0±13.0) and RA (34.1±9.8) subjects. Our findings are consistent with the order of impairment reported (with SSc having worst, RA intermediate and SLE least impairment in PCS) and extend the results to IIM, which we found to have the most severe impairment in physical HRQoL. In addition, our data indicate that the order of impairment in physical HRQoL is not the same as in mental HRQoL, with SLE having at once less impairment in the former and more in the latter compared to RA and SSc. This cross-disease comparison thus contributes important novel findings in HRQoL in SARD.

The differences in selected clinical features between diseases provide some insight into the differences in HRQoL that we found. Indeed, early RA is predominantly a single organ disease, whereas IIM is clearly multi-systemic. On the other hand, the cross-sectional design of this study did not allow us to study the effect of disease course and treatment on HRQoL. SLE is characterized by intermittent flares whereas SSc can have a more progressive course, with some subsets rapidly progressing and others progressing at a more indolent rate. Also, RA, IIM and SLE are generally responsive to glucocorticoids and systemic disease-modifying drugs, whereas treatment options in SSc are currently limited. These outstanding questions provide impetus to further understand the commonalities and differences in the predictors of HRQoL and to develop common and tailored interventions to improve HRQoL in these serious, chronic diseases.

This study is not without limitations. First, HRQoL was measured using various versions of the Medical Outcomes Trust Short Form questionnaires. The SLE cohort used version 1 of the SF-36 questionnaire, the SSc and IIM subjects used version 2 of the SF-36 questionnaire, and RA subjects used the SF-12. Nevertheless, the SF-12 has been shown to be a reliable measure of quality of life and comparable to the SF-36 with only slight loss of performance. Also, both the PCS and MCS scores of the SF-12 were shown to correlate strongly with those of the SF-36 [12, 21] and scores from version 1 and version 2 of the SF-36 have also been shown to be comparable due to a re-estimation of norms for version 1 upon the release of version 2 [13]. Second, the harmonized variables in our cross-disease dataset usually used lowest common denominators. This may have resulted in a loss of precision in the definition of the covariates in our models. Third, the worst results in IIM might have been due to differences in recruitment and sampling; that is, we may have recruited only the sickest IIM patients, and a broader spectrum of the SSc, RA, and SLE population. However, that is unlikely since IIM subjects were recruited from the same tertiary centers as the other disease groups. Fourth, the sample of some of the disease groups, in particular IIM, was small. This also contributed to poor precision. Finally, although fibromyalgia may impact HRQoL, none of the study cohorts collected data on this. Hence, its impact on HRQoL could not be determined in this study. Thus, at this time, our results should be interpreted with caution. Larger studies will be required to confirm the findings reported in this study. Nevertheless, the strength of the study lies in our unique ability to compare 4 separate SARDs with incident subjects using a harmonized dataset.

## Conclusion

There is currently a paucity of HRQoL data in SARD in early disease. This research was designed to fill this important knowledge gap. We found that SARD type was an independent predictor of HRQoL, with differences that were clinically meaningful. In particular, IIM subjects, for whom there is the least published data on HRQoL, had the most severe impairment in both physical and mental HRQoL. Cross-SARD research provides a novel approach to gain greater understanding of commonalities and differences across rheumatic diseases. The differences in baseline HRQoL across incident SARD provide impetus to pursue studies to identify the common and distinct predictors of HRQoL in early SARD as well as to determine trajectories of HRQoL over time. This knowledge will guide the development of common and tailored interventions to improve HRQoL in these serious, chronic diseases.

## Supporting information

S1 File Details of the research cohorts and study variables. (DOCX)
