# Supplementary material for: A comparison of health-related quality of life (HRQoL) across four systemic autoimmune rheumatic diseases (SARDs)
Source: PLoS One. 2017 Dec 19;12(12):e0189840. doi: 10.1371/journal.pone.0189840 (PMC5736192; doi:10.1371/journal.pone.0189840)
Supplement: S1 File — (DOCX) [file pone.0189840.s001.docx]

**Supporting Information**

**S1 File. Details of the research cohorts and study variables.**

***Study Subjects***

The study subjects consisted of those enrolled in the Canadian Scleroderma Research Group (CSRG), the McGill University Health Centre (MUHC) Systemic Lupus Erythematosus (SLE) cohort, the Canadian Early Arthritis Cohort Study (CATCH), and the Canadian Inflammatory Myopathy Study (CIMS) cohorts.

Subjects enrolled in the CSRG registry are recruited from 15 centers across Canada. They must have a diagnosis of SSc made by a rheumatologist, be ≥18 years of age and be fluent in either French or English. Over 98% of the cohort meets the 2013 ACR/EULAR classification criteria for systemic sclerosis^1^.

The MUHC SLE cohort consists of a single center registry of SLE patients who fulfill the revised American College of Rheumatology (ACR) criteria for SLE ^2,3^ and are able to provide informed consent in French or English.

Patients enrolled in the CATCH registry are recruited from 19 sites across Canada. To be included, patients must be > 16 years of age, have joint symptoms for more than 6 weeks but less than 12 months and have at least one of the following: ≥2 swollen joints, one swollen metacarpophalangeal, or one proximal interphalangeal joint. Patients must also have one or more of the following: positive rheumatoid factor, positive anti-cyclic citrullinated peptide antibody, morning stiffness for more than 45 minutes, response to nonsteroidal antiinflammatory drugs, or a positive metatarsophalangeal squeeze test.

Subjects enrolled in the CIMS multi-site registry must have onset of symptoms attributable to their myopathy of < 3 years duration, be ≥18 years of age and be fluent in either French or English. Classification criteria for IIM date back almost 35 years to initial publications by Medgser et al.^4^, and now include no less than 10 sets, each with advantages and disadvantages^5^. An ACR/EULAR collaboration is in fact underway to develop new classification criteria for IIM^5^. In the meantime, CIMS has taken a broad view of IIM and includes subjects who meet the original PM/DM criteria according to the Bohan and Peter^6,7^, clinically amyopathic dermatomyositis criteria according to Gerami et al.^8^, necrotizing myositis according to Amato and Barohn^9^ and/or overlap myositis proposed by Troyanov et al.^10^

Ethics committee approval for each research cohort was obtained at McGill University (Montreal, Canada) and at all participating sites. All subjects provided informed written consent to participate in the data collection protocol of the relevant research cohort.

***Sociodemographic Variables***

The sociodemographic variables (age, gender, race/ethnicity, education, smoking exposure) were self-reported by the study subjects.

*Race/Ethnicity***:**

In the CSRG and CIMS protocols, race/ethnicity was defined as White, Chinese, South Asian (eg. East Indian, Pakistani, Sri Lankan, etc), Black, Filipino, Latin American, Southeast Asian (eg. Cambodian, Indonesian, Laotian, Vietnamese, etc), Arab, West Asian (eg. Afghan, Iranian, etc), Japanese, Korean, French Canadian (defined as 4 francophone grand-parents born in the province of Quebec), or Aboriginal (North American Indian, Métis, Inuit, Indian/First Nation). Patients reported as many ethnicities as applied. In the MUHC SLE protocol, race/ethnicity was defined as Aboriginal, Arab/Middle Eastern, Asian, Black, Jewish, Latin/Hispanic, Pacific Islander, White. Patients reported all applicable ethnicities. In CATCH, race/ethnicity was defined as Caucasian, Hispanic, African Canadian, Native Canadian, European, South Asian/Indian, or East Asian/Oriental. Patients reported as many as applied. The harmonized definition of race/ethnicity used for comparison across the 4 cohorts was: White, Black, Asian, Hispanic, Arab, French Canadian, and Aboriginal.

*Education:*

In the CSRG and CIMS protocols, education was defined as the highest level of education received in 8 categories: less than seven years of school, grades seven through nine, grades ten through eleven, high school graduate, one to four years of CEGEP/college or university, CEGEP/college graduate, university graduate, and post-graduate education. In the MUHC SLE cohort, patients were asked to report how many years of schooling they had completed, from 0 to 17. In CATCH, education was defined as the highest level received in one of 6 categories: elementary school, high school, college/trade school, university/bachelor, master, or PhD. The harmonized definition used was the highest level of education received in one of 3 categories: elementary, high school, and college/trade school/university.

*Smoking Exposure:*

In the CSRG, CIMS and MUHC SLE protocols, smoking exposure was divided into 3 categories: never, in the past only, and current smoker. In CATCH, smoking exposure was defined as smoking or chewing tobacco and it was divided into 3 categories: never, current, and ex-smoker. The harmonized definition was divided into 3 categories: never smoked (never smoked or chewed tobacco), current smoker (currently smokes or chews tobacco) and past smoker (only smoked or chewed tobacco in the past, not now).

***Disease Variables***

*Disease duration:*

Disease duration was recorded by study physicians in all 4 cohorts. In the CSRG cohort, disease onset was defined as the onset of the first non-Raynaud’s disease manifestation (i.e. skin tightening, shortness of breath, dyspepsia, etc.). Disease onset in CIMS was defined as the date when the patient noticed the first symptoms attributable to IIM (i.e. including but not limited to muscle weakness, muscle pain or rash). In the SLE cohort, disease onset was defined as the date when the subject accumulated 4 or more ACR classification criteria for SLE. In CATCH, disease duration was assessed since the onset of symptoms (i.e. persistent pain, swelling or stiffness). The harmonized definition for disease duration was the date of the baseline study visit minus the date of disease onset.

*Lung disease:*

In the CSRG registry, the presence of interstitial lung disease was determined using a clinical decision rule that was recently published ^11^. Using this algorithm, interstitial lung disease was considered present if a high resolution computed tomography (HRCT) scan of the lung was interpreted by an experienced radiologist as showing interstitial lung disease or, in the case where no HRCT is available, if either a chest x-ray was reported as showing either increased interstitial markings (not thought to be due to congestive heart failure) or fibrosis, and/or if a study physician reported the presence of typical “velcro-like crackles” on physical examination. In the CIMS cohort, interstitial lung disease was considered present if a study physician reported the presence of active reversible interstitial lung disease on the Myositis Disease Activity Assessment Tool (MDAAT) and/or pulmonary fibrosis on the Myositis Damage Index (MDI)^12^. In the MUHC SLE subjects, interstitial lung disease was present if a physician reported the presence of pulmonary fibrosis on the System Lupus International Collaborating Clinics/ACR Damage Index^13^. In CATCH, participating physicians report the presence of extra-articular features, including interstitial lung disease, in the physician case report form.

*Raynaud’s disease:*

The presence of Raynaud’s phenomenon was reported by study physicians in all four study cohorts.

*Sicca:*

The presence of ‘sicca’ was patient-reported in the CSRG, CIMS and CATCH protocols. In the CSRG and CIMS protocols, study subjects are asked to report yes/no to the presence of daily, persistent, troublesome dry eyes for more than three months and/or a feeling of dry mouth on a daily basis for more than three months. Subjects in CATCH report the regular presence of dry mouth in the past month. The MUHC SLE protocol does not record the presence of sicca.

*Arthritis:*

‘Arthritis’ was defined as follows: in the CSRG, if the study physician reported a history of inflammatory polyarthritis or overlap with rheumatoid arthritis; in the MUHC SLE subjects if the study physician reported arthritis as one of the ARA classification criteria for SLE or if objective synovitis was noted to be present on the SLEDAI-2K^14^; in CIMS, if active polyarthritis (whether mild, moderate or severe) was noted to be present on the MDAAT^12^ or if the study physician reported the presence of overlap with rheumatoid arthritis. By definition, all subjects in CATCH were considered to have arthritis.

*Myositis:*

The variable ‘myositis’ was MD-reported in the CRSG protocol, with physicians answering yes/no to the question “now or in the past, has the patient had inflammatory myositis”. In CIMS, all patients were reported as having myositis, except those who were reported to have amyopathic disease by a study physician (defined as no symptoms of weakness and a normal creatine kinase value). In the MUHC SLE subjects, myositis was considered present if a physician reported its presence in the preceding 10 days on the SLEDAI-2K^14^. In CATCH, participating physicians report the presence of extra-articular features, including myositis, in the physician case report form.

*Global disease activity:*

‘Global Disease Activity’ was physician-reported in all 4 cohorts. In the CSRG, a numerical rating scale ranged from 0 (no disease activity) to 10 (most activity). In CIMS, a numerical rating scale ranged from 0 (no disease activity) to 10 (very severe disease). In the MUHC SLE protocol, physicians used a visual analogue scale ranging from 0 (no activity) to 10 (most activity). Finally, in CATCH, physicians used a visual analogue scale ranging from 0 (not active) to 10 (very active). Activity of the disease was assessed at the time of the visit (SLE and CATCH), during the previous week (CSRG) and in the last month (CIMS).

*Global disease damage:*

‘Global Disease Damage’ was physician reported in the CSRG (numerical rating scale ranging from 0 (no damage) to 10 (most damage), CIMS (numerical rating scale ranging from 0 (absent) to 10 (maximum) and MUHC SLE protocols (visual analogue scale ranging from 0 (no damage) to 10 (most damage)). Damage was assessed at the time of the visit (SLE and CIMS) and during the previous week (CSRG). Data on disease damage was not available for the CATCH subjects.

***Outcome variables***

The MUHC SLE cohort collected HRQoL data using version 1 of the Medical Outcomes Trust Short Form 36 (SF-36) while the CSRG and CIMS cohorts used version 2. CATCH used version 2 of the Medical Outcomes Trust Short Form 12 (SF-12). All forms and versions of the questionnaire can be summarized into Physical Component Summary (PCS) and Mental Component Summary (MCS) scores, which have been shown to be directly comparable ^15,16^.

***References***

1. Alhajeri H, Hudson M, Fritzler M, Pope J, Tatibouet S, Markland J, et al. 2013 American College of Rheumatology/European League against rheumatism classification criteria for systemic sclerosis outperform the 1980 criteria: data from the Canadian Scleroderma Research Group. Arthritis Care Res (Hoboken). 2015;67(4):582-7. doi: 10.1002/acr.22451. PubMed PMID: 25233870.

2. Tan EM, Cohen AS, Fries JF, Masi AT, McShane DJ, Rothfield NF, et al. The 1982 revised criteria for the classification of systemic lupus erythematosus. Arthritis Rheum. 1982;25(11):1271-7. PubMed PMID: 7138600.

3. Hochberg MC. Updating the American College of Rheumatology revised criteria for the classification of systemic lupus erythematosus. Arthritis Rheum. 1997;40(9):1725. doi: 10.1002/1529-0131(199709)40:9&lt;1725::AID-ART29&gt;3.0.CO;2-Y. PubMed PMID: 9324032.

4. Medsger TA, Jr., Dawson WN, Jr., Masi AT. The epidemiology of polymyositis. Am J Med. 1970;48(6):715-23. PubMed PMID: 5420557.

5. Mahler M, Miller FW, Fritzler MJ. Idiopathic inflammatory myopathies and the anti-synthetase syndrome: a comprehensive review. Autoimmun Rev. 2014;13(4-5):367-71. doi: 10.1016/j.autrev.2014.01.022. PubMed PMID: 24424190; PubMed Central PMCID: PMCPMC3970575.

6. Bohan A, Peter JB. Polymyositis and dermatomyositis (first of two parts). N Engl J Med. 1975;292(7):344-7. doi: 10.1056/NEJM197502132920706. PubMed PMID: 1090839.

7. Bohan A, Peter JB. Polymyositis and dermatomyositis (second of two parts). N Engl J Med. 1975;292(8):403-7. doi: 10.1056/NEJM197502202920807. PubMed PMID: 1089199.

8. Gerami P, Schope JM, McDonald L, Walling HW, Sontheimer RD. A systematic review of adult-onset clinically amyopathic dermatomyositis (dermatomyositis sine myositis): a missing link within the spectrum of the idiopathic inflammatory myopathies. J Am Acad Dermatol. 2006;54(4):597-613. doi: 10.1016/j.jaad.2005.10.041. PubMed PMID: 16546580.

9. Amato AA, Barohn RJ. Evaluation and treatment of inflammatory myopathies. J Neurol Neurosurg Psychiatry. 2009;80(10):1060-8. doi: 10.1136/jnnp.2008.169375. PubMed PMID: 19762898.

10. Troyanov Y, Targoff IN, Tremblay JL, Goulet JR, Raymond Y, Senecal JL. Novel classification of idiopathic inflammatory myopathies based on overlap syndrome features and autoantibodies: analysis of 100 French Canadian patients. Medicine (Baltimore). 2005;84(4):231-49. PubMed PMID: 16010208.

11. Steele R, Hudson M, Lo E, Baron M, Canadian Scleroderma Research G. Clinical decision rule to predict the presence of interstitial lung disease in systemic sclerosis. Arthritis Care Res (Hoboken). 2012;64(4):519-24. doi: 10.1002/acr.21583. PubMed PMID: 22213733.

12. Rider LG, Werth VP, Huber AM, Alexanderson H, Rao AP, Ruperto N, et al. Measures of adult and juvenile dermatomyositis, polymyositis, and inclusion body myositis: Physician and Patient/Parent Global Activity, Manual Muscle Testing (MMT), Health Assessment Questionnaire (HAQ)/Childhood Health Assessment Questionnaire (C-HAQ), Childhood Myositis Assessment Scale (CMAS), Myositis Disease Activity Assessment Tool (MDAAT), Disease Activity Score (DAS), Short Form 36 (SF-36), Child Health Questionnaire (CHQ), physician global damage, Myositis Damage Index (MDI), Quantitative Muscle Testing (QMT), Myositis Functional Index-2 (FI-2), Myositis Activities Profile (MAP), Inclusion Body Myositis Functional Rating Scale (IBMFRS), Cutaneous Dermatomyositis Disease Area and Severity Index (CDASI), Cutaneous Assessment Tool (CAT), Dermatomyositis Skin Severity Index (DSSI), Skindex, and Dermatology Life Quality Index (DLQI). Arthritis Care Res (Hoboken). 2011;63 Suppl 11:S118-57. doi: 10.1002/acr.20532. PubMed PMID: 22588740; PubMed Central PMCID: PMCPMC3748930.

13. Gladman D, Ginzler E, Goldsmith C, Fortin P, Liang M, Urowitz M, et al. The development and initial validation of the Systemic Lupus International Collaborating Clinics/American College of Rheumatology damage index for systemic lupus erythematosus. Arthritis Rheum. 1996;39(3):363-9. PubMed PMID: 8607884.

14. Gladman DD, Ibanez D, Urowitz MB. Systemic lupus erythematosus disease activity index 2000. J Rheumatol. 2002;29(2):288-91. PubMed PMID: 11838846.

15. Hurst NP, Ruta DA, Kind P. Comparison of the MOS short form-12 (SF12) health status questionnaire with the SF36 in patients with rheumatoid arthritis. Br J Rheumatol. 1998;37(8):862-9. PubMed PMID: 9734677.

16. Ware J, Kosinski M, Dewey J. How to score version 2 of the SF-36 Health Survey (standard and acute forms). Lincoln, R.I.: QualityMetric, Inc.; 2005.
